# Medical and Philosophical Intelligence

**Published:** 1812-10

**Authors:** 


					too
MEDtCAL ams PHILOSOPHICAL INTELLIGENCE;
GLASGOW MEDICAL SCHOOL.?The following Annual
Prelections will commence in the first week of November next,
and continue till May.
Anatomy and Surgery.?Dr. Jeffrey, University; Mr. Allan
Burns, College-street.
Practice of Medicine.?Dr. Freer, University; Dr. Robert Walt,
College-street.
Theory of Medicine.?Dr. Freer, University; Dr. Robert Watt,
College-street.
Materia Medica.?Dr. Millar, University; Dr. Ure, Anderson's
Institution.
Chemical and Medical Pharmacy.?Dr. Cleghorn, University;
Dr. Ure, Anderson's Institution.
Midwifery, and Disease* of Women, Sfc.?Mr. Towers, Univer-
sity; Mr. John Burns, College-street.
Clinical Lectures.?Dr Robert Cleghorn, and Dr. Richard Millar,
Clinical Surgery.?Mr. John Scrutore.
Veterinary Medicine and Surgery.?Mr. Courer.
From the above statement it will be seen, that students resorting
to Glasgow may have a choice of Lectures in every branch of the
profession. They have also the benefit of an extensive and well-
regulated Infirmary, and admission to the Museum of the late
celebrated Dr. Wijiiam Hunter.
The Faculty of Physicians and Surgeons of Glasgow, have enacted
the following Laws respecting Surgeons'Diplomas:?Every candi-
date for a diploma must, previously to his being taken on *rial,
produce satisfactory evidence that he has studied medical science
three complete winter sessions, either in an university, or under
members of the colleges of physicians and surgeons of Edinburgh,
London, Dublin, or under members of the faculty of physicians
and surgeons of Glasgow, the following public courses of lectures,
namely, two courses of anatomy and surgery, delivered in different
sessions; one course of chemistry; one of materia medica; one of
the theory of medicine; one 011 the practice of medicine; and one
011 midwifery; and that he has attended one year in a public hospi-
tal, and studied practical pharmacy in a regular surgeon's or apo-
thecary's shop during at least six months. Candidates who have
served a regular apprenticeship of three or more years to a regular
practitioner, must produce evidence of having attended all the above
classes and a public hospital during one year; but the duration of
their studies may be abridged to two winter sessions.
Faculty Hall, Glasgow, Sept. 10, IS 12.
Dr. Adams's autumnal course of Lectures on the Institutes and
Practice of Medicine, will commence on Thursday the 8th of Oc-
tober, at ten o'clock precisely, at his house, No. 17, Hatton Gar-
den, and to be continued <??ery succeeding Tuesday, Thursday, and
Saturday, at the same hour,
'" Medical
Medical and Philosophical Intelligence.
Medical and Chemical Lectures and Examinations.?Dr. Hooper
4nd Dr. Agar will begin their next course on the Theory and Prac-
tice of Physic, Chemistry, and the Materia Medica, at the Theatre,
20", Cork-street, Burlington Gardens, on Monday, Octobers, 1812,
at eight o'clock in the morning. Three courses are given yearly, be^
ginning in February, June, and October, each on the first Monday
of the month.
Lectures on Surgery, Physiology, and Pathology.?Mr. A. Car-
lisle, F.It.S. 1'rofessor of Anatomy in the Royal Academy, and Sur-
geon of the Westminster Hospital, will begin his course of Lectures
on the Art and Practice of Surgery, and the Sciences connected
therewith, on Monday, October 12, at half after eight, P.M. at
his house in Soho Square. The introductory discourse is open to
all professional students, and the subject to be continued on Mon-
days, Wednesdays, and Fridays, at the same hours. The diseases
and accidents allotted to the province of surgery will be amply
treated bf, and illustrated by cases from the lecturer's experience.
A compendious view of the animal economy will be adduced to illus-
trate the several processes of disease, and of recovery. The opera-
tions of surgery, and the anatomy of the affected parts, are to be
demonstrated.
Theatre of Anatomy, Blenheim-Street, Great Marlborough-
Street.?The autumnal course of Lectures on Anatomy, Physiology,
and Surgery, will be commenced on Thursday, the 1st of October,
at two o'clock, by Mr. Brookes. Anatomical Converzatiofies will
be held weekly, when the different subjects treated of will be dis-
cussed familiarly, and the students' views forwarded. To these
none but pupils can be admitted. Spacious apartments, thoroughly
ventilated, and replete with every convenience, are open all the
inorning, for the purposes of dissecting and injecting, where Mr.
Brookes attends to diiect the students, and demonstrate the various
parts as they appear on dissection. The inconvenience's usually
attending anatomical investigations, are counteracted by an anti-
septic process. Pupils may be accommodated in the house. Gen-
tlemen established in practice desirous of renewing their anatomical
knowledge, may, be accommodated with an apartment to dissect in
privately.
Dr. Squires Lectures.~T)ic. Squire will, on Tuesday, October 6,
begin a course of Lectures on the Theory and Practice oi Midw ifery,
and the Diseases of Women and Children.
Dr. Ramsbotham will commence his first winter course of Lec-
tures on the Science and Practice of Midwifery, on Monday, Octo-
ber 5, at his House, No. 9, Old Jewry.
y y 2 St.
j j
St. Georges Hospital, and George Street, Hanover Square,
Medical and Chemical Lectures.?On Monday, October 5, a course
of Lectures on Physic and Chemistry, will re-commence at No. y,
George Street, Hanover Square, at the usual morning hours, vi/.
the Medical Lectures at eight, and the Chemical at a quarter after
nine o'clock, by George Pearson, M.D. F.R S. Senior Physician to
St. George's Hospital, Physician in Ordinary to their Royal High-
nesses the Duke and Duchess of York, and to their Household ;
Honorary Fellow of the Imperial Medico-Chirurgical Academy at
St. Petersburg!), of the College of Physicians, &c. Ac.
Note, Clinical Lectures are given on the cases of patients in
St. George's Hospital, every Saturday morning, at nine o'clock.
Weekly Return of Deaths by Small-pox, from the London Bills of
Mortality-
July 14, .... 25
21, .... 24
28, . ... 26
Aug. 4, . . . 26
Aug. J], ; . . 21
18, .... 61
25 30
Sept. 1, .... 32

				

